# Temporal Trends in the Chronobiology and Epidemiology of Sepsis Admissions over 21 Years: A Nationwide Study in Northwestern Spain

**DOI:** 10.3390/antibiotics15060579

**Published:** 2026-06-07

**Authors:** David Andaluz Ojeda, María Paz Barrio Alonso, Iván Cusácovich Torres, José Ramón Garmendia Leiza, Ángela González Salamanca, Francisco José Manuel Merino, Leire Pérez Bastida, Alberto Pérez Rubio, Laura Sanz Rueda, Jesús María Andrés de Llano

**Affiliations:** 1 Department of Intensive Care Medicine, Complejo Asistencial Universitario de Palencia, 34005 Palencia, Spain; dandaluz@saludcastillayleon.es (D.A.O.); agonzalezsal@saludcastillayleon.es (Á.G.S.); fjmanuel@saludcastillayleon.es (F.J.M.M.); leireperezb@saludcastillayleon.es (L.P.B.); 2 BioCritic, Group for Biomedical Research in Critical Care Medicine, Universidad de Valladolid, 47005 Valladolid, Spain; 3 Department of Paediatrics, Hospital Clínico Universitario de Valladolid, 47003 Valladolid, Spain; 4 Department of Internal Medicine, Hospital Clínico Universitario de Valladolid, 47003 Valladolid, Spain; icusacovich@saludcastillayleon.es; 5 Health Centre Jardinillos, Gerencia de Salud de Área de Palencia, 34001 Palencia, Spain; jgarmendia@saludcastillayleon.es; 6 Hospital Nuestra Señora de Sonsoles de Ávila, 05004 Ávila, Spain; aperezru@saludcastillayleon.es; 7 Health Centre Villamuriel de Cerrato, Gerencia de Salud de Área de Palencia, 34190 Palencia, Spain; lsanzr@saludcastillayleon.es; 8 Department of Paediatrics, Complejo Asistencial Universitario de Palencia, 34005 Palencia, Spain; jandresl@saludcastillayleon.es

**Keywords:** epidemiology, chronobiology, sepsis, mortality

## Abstract

Background: Sepsis remains one of the leading causes of hospital morbidity and mortality worldwide. While its epidemiology has been extensively investigated, the chronobiology of sepsis—the temporal patterns underlying its occurrence and outcomes—has received comparatively little attention, despite its potential relevance for anticipating clinical demand and optimizing healthcare resource allocation. Objective: To analyze trends in the epidemiology and chronobiology of hospital admissions for sepsis across a large healthcare region in Spain over a 21-year period. Design: Retrospective observational study based on clinical–administrative records. We included all patients ≥ 18 years who had a principal diagnosis of sepsis or septic shock admitted between 2001 and 2021, identified through ICD-9-CM and ICD-10-CM coding in the Spanish National Hospital Discharge Records Database. Annual incidence, in-hospital mortality, and temporal distribution of admissions and deaths were assessed. Joinpoint regression was used to evaluate trends, Fourier spectral analysis to identify dominant rhythms, and multi-harmonic cosinor models to test for circannual rhythmicity. Results: We identified 39,622 sepsis admissions. Hospital incidence increased significantly over time (annual percent change [APC]: +9.6%; *p* < 0.001), with two inflection points (2008 and 2014). In-hospital mortality decreased linearly from 42.1% in 2001 to 31.9% in 2021 (*p* < 0.001), despite a progressive increase in patient age. Chronobiological analyses revealed no significant circannual rhythm in incidence or mortality (cosinor *p* = 0.14), although mortality was disproportionately clustered in winter months (*p* < 0.001). In multivariable analyses, clinical and epidemiological variables were independent predictors of in-hospital mortality. Conclusions: Over the last two decades, sepsis incidence has risen steadily, whereas hospital mortality has declined. Although no regular biological rhythm was demonstrated, excess winter mortality suggests extrinsic seasonal influences. This study provides novel evidence by jointly examining the epidemiology and chronobiology of sepsis, and supports their integration into healthcare planning strategies.

## 1. Introduction

Sepsis is defined as life-threatening organ dysfunction caused by a dysregulated host response to infection [1]. It represents a primary public health concern, with an estimated annual incidence of around 50 million cases worldwide, affecting all age groups but particularly prevalent at the extremes of age and in states of immunosuppression [2,3,4]. Since the first consensus definition published in 1991 by Bone et al., the incidence of sepsis and septic shock has continued to rise. This trend is attributed to factors such as population aging—especially in Western countries—greater use of invasive procedures, the emergence of multidrug-resistant bacteria, and the widespread use of immunosuppressive or chemotherapeutic treatments [5,6]. Sepsis therefore constitutes a major public health challenge. A recent study estimated that, on average, healthcare expenditure related to sepsis represents 0.33% of a country’s gross national product, with mortality rates ranging from 15% to 40% depending on the series [2,7]. Moreover, survivors frequently experience long-term physical, psychological, and cognitive sequelae with significant social and healthcare implications, and face an increased risk of readmission and late mortality [8].

Because of this high morbidity and mortality, recent years have seen major efforts to raise awareness and train healthcare professionals in the early recognition and timely management of sepsis. Initiatives such as the Surviving Sepsis Campaign (SSC) and the creation of multidisciplinary in-hospital “Sepsis Code” teams have aimed to facilitate early identification and the implementation of bundled measures, prompt antibiotic therapy, fluid resuscitation, and vasopressor use, all of which are critical for improving outcomes in this time-dependent disease [9,10,11,12,13].

Despite these initiatives, the global epidemiology of sepsis remains poorly characterized. Variables such as incidence, mortality, and clinical presentation may have been influenced by evolving case definitions, the introduction of new diagnostic and therapeutic strategies promoted by campaigns such as the SSC, and primary events including the rise in antimicrobial resistance and the SARS-CoV-2 pandemic. In addition, the chronobiology of sepsis—that is, the study of temporal patterns in its occurrence and outcomes—has received little attention, despite its potential utility for optimizing resource allocation and anticipating peaks in healthcare demand.

Chronobiological analysis is a useful tool for efficient planning of human and material resources in healthcare systems, as it allows identification of temporal patterns in incidence, hospital admissions and mortality. This information can help optimize healthcare organizations and implement strategies aimed at improving care and reducing avoidable mortality. In sepsis, seasonal variation is plausible and may relate to changes in the epidemiology of underlying infections: respiratory infections may predominate in colder months, whereas gastrointestinal infections may be more common in warmer months.

In the present study, we aim to analyze epidemiological trends and chronobiological patterns of sepsis in Castilla y León, a large region in northwestern Spain, over the past two decades using the National Hospital Discharge Database (NHDS). The ultimate goal is to provide updated information that can inform future strategies for prevention, diagnosis, and clinical management.

## 2. Results

Between January 2001 and December 2021, a total of 4,480,200 hospital admissions involving patients older than 18 years were recorded in the public hospital network of Castilla y León. Of these, 39,622 (0.88%) had a principal diagnosis of sepsis or septic shock at admission.

The overall mean incidence of sepsis in the population was 70 cases per 100,000 inhabitants per year, with a significant annual increase from 18 cases per 100,000 in 2001 to 165 cases per 100,000 in 2021.

### 2.1. Demographic Characteristics, Incidence, and Epidemiology

Among all patients admitted with a diagnosis of sepsis, 55.3% were male, 46.8% came from rural areas (settlements with fewer than 10,000 inhabitants), 55% of admissions occurred in tertiary hospitals, 38% in secondary hospitals, and only 6.7% were admitted to community hospitals (level 1).

**Figure 1:**
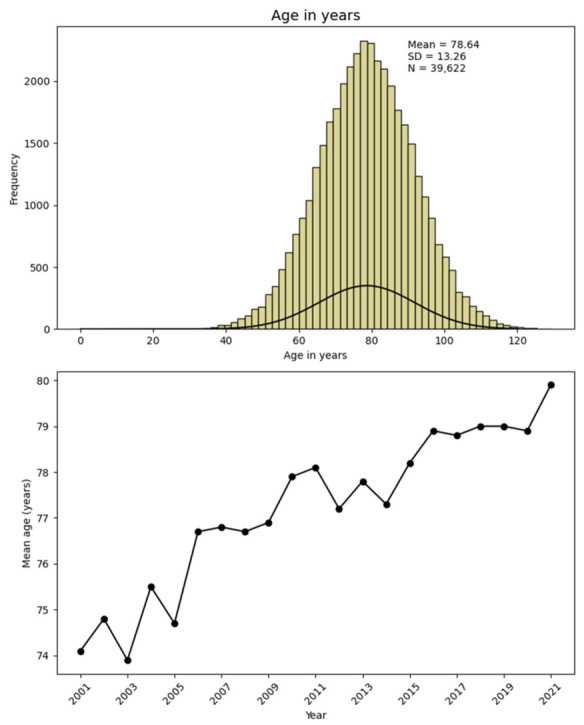
Distribution of patients by age at admission (**upper panel**) and mean age (years) at admission throughout the study period (**lower panel**).

The mean age of the patients was remarkably high, 78.6 ± 13.26 years, and showed an increasing trend throughout the study period. The mean age at admission rose from 74.7 ± 15.01 years in 2001 to 78.6 ± 13.26 years in 2021 (*p* < 0.001), as shown in Figure 1.

The main comorbidities among patients included were previous heart disease in 44% of cases, hypertension in 28.6%, diabetes in 24.8%, and dyslipidemia in 17.6%. Chronic kidney disease was present in 14%, chronic respiratory disease/COPD in 12%, and a history of neoplastic disease in 16.9% of patients (Table 1).

**Figure 2:**
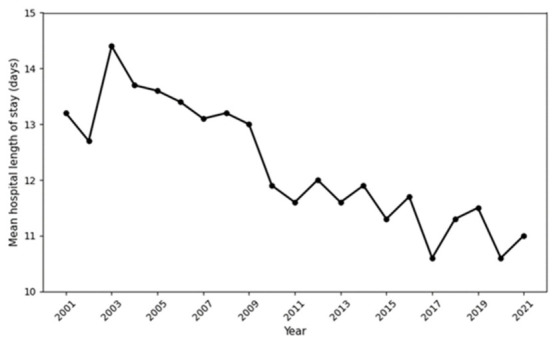
Mean hospital length of stay (days) over the study years.

The mean hospital length of stay was 11.58 ± 11.94 days, with a significantly decreasing trend over time: from 13.18 ± 13.35 days in the first year of the study to 10.07 ± 10.21 days in the last year (*p* < 0.001), as shown in Figure 2.

Regarding etiology, an etiological microorganism was identified in only 40% of cases. Among these, 74% were Gram-negative bacteria and 23% Gram-positive bacteria, with the most frequently documented organisms being *E. coli* (34.8%), *Staphylococcus* spp. (9.2%), and *Klebsiella* spp. (7.6%). Other identified pathogens included fungi and viruses, predominantly *Candida* spp. (93.4% of fungal isolates) and influenza virus among viral infections. Regarding organ dysfunction, cardiovascular involvement (shock) was observed in 72.7% of patients, renal dysfunction in 72%, respiratory involvement in 51%, digestive tract involvement in 34.5%, and neurological involvement in 22% (Table 1).

### 2.2. Chronobiology and Trends

Over the 21-year study period, we observed a linear and statistically significant increase in the annual rate of hospital admissions for sepsis, rising from 380 cases in 2001 to 4164 cases in 2021. As mentioned above, we also observed an increase in the mean age of patients admitted with sepsis over the course of the study. To address this potential bias, we performed a joinpoint linear regression analysis, stratifying incidence rates by age groups.

**Figure 3:**
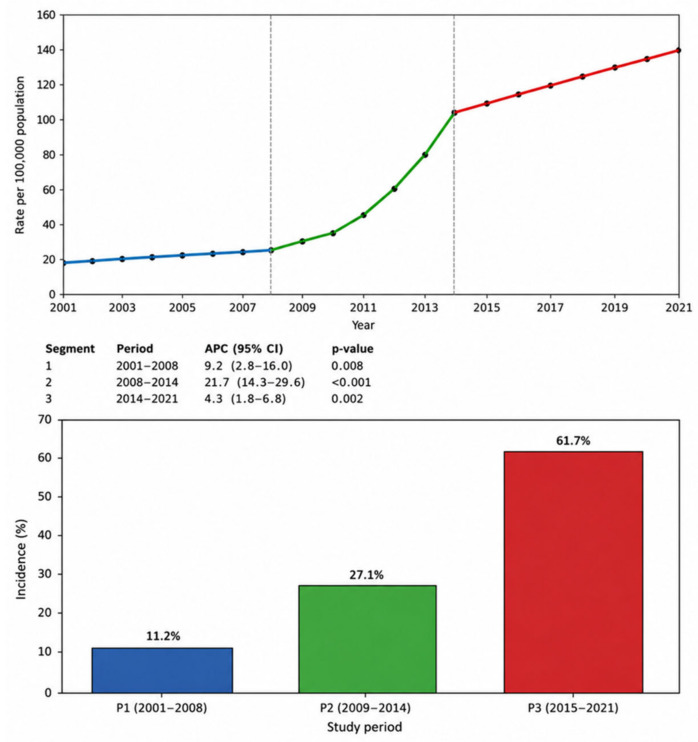
Upper panel: Log-linear joinpoint analysis assessing the rate of hospitalized patients with a principal diagnosis of sepsis among adults over 18 years of age from January 2001 to December 2021 (Blue = Period 1; Green = Period 2; Red = Period 3). Lower panel: Cumulative incidence of sepsis by study period.

Joinpoint regression analysis identified two statistically significant inflection points, in 2008 and 2014 (*p* < 0.05). The annual percentage change (APC) was 9% (95% CI 2.4–16.0, *p* = 0.01) for the period 2001–2008 (P1), 21.8% (95% CI 14.6–29.5, *p* < 0.001) for 2008–2014 (P2), and 4.4% (95% CI 1.9–6.9; *p* = 0.002) for 2014–2021 (P3), as shown in Figure 3.

Spectral analysis using the Fast Fourier Transform (FFT) was performed to identify dominant periodic components, followed by cosinor analysis with multiple harmonics to assess the presence of a circannual rhythm. No consistent seasonal pattern was identified (*p* = 0.14). Thus, no seasonal increase in sepsis admissions was observed, with incidence trends remaining similar throughout the months of the year.

### 2.3. Sepsis Mortality During the Study Period

#### 2.3.1. Patients and Hospitals

The overall in-hospital mortality in the study population was 35.7%. Age was one of the main factors associated with mortality: the mean age of deceased patients was significantly higher than that of survivors (82.1 vs. 76.8 years, *p* < 0.001). Mortality was particularly high among patients over 75 years compared with those younger than 75 (40.9% vs. 24.4%, *p* < 0.001). Of the 14,152 deceased patients in our cohort, 78.6% were aged 75 years or older.

Although men represented the majority of patients admitted with sepsis, mortality was slightly lower in men than in women (34.3% vs. 37.4%; *p* < 0.001). Deceased patients also had a shorter mean length of stay compared with survivors (7.9 vs. 13.6 days, *p* < 0.001).

**Figure 4:**
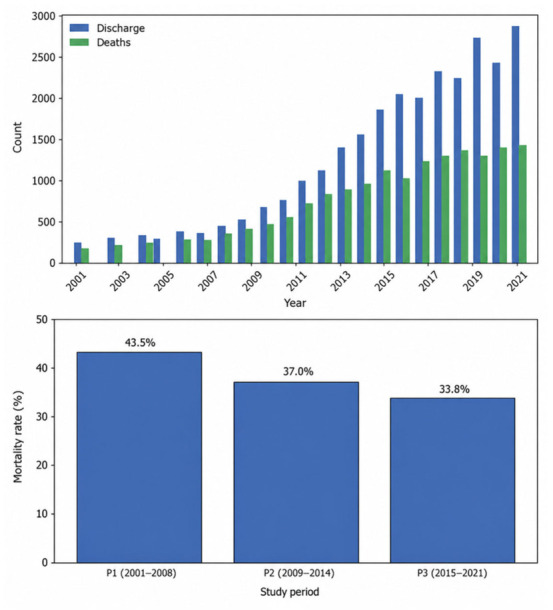
Annual trends in cumulative hospital mortality over the study period (**upper panel**) and stratification by joinpoint-defined periods (**lower panel**).

Among deceased patients, 51.6% came from urban areas and 48.4% from rural areas (*p* < 0.001). However, mortality rates were higher in rural areas (36.9%) compared with urban areas (34.7%; *p* < 0.001). Mortality was higher among patients admitted to level-2 hospitals (37.5%) compared with level 1 (34.7%) and level 3 hospitals (34.6%) (*p* < 0.001) (Figure 4). Mortality was also higher in patients classified under medical DRGs (Diagnosis-Related Groups) (36.2%) compared with surgical DRGs (27.8%) (*p* < 0.001) (Supplementary Figure S1).

No significant differences were observed in mortality according to admission type (urgent vs. elective, *p* = 0.26). Internal Medicine accounted for the highest percentage of in-hospital deaths (72.5%), followed by Intensive Care Medicine (9.2%).

#### 2.3.2. Chronobiology and Mortality Trends

**Figure 5:**
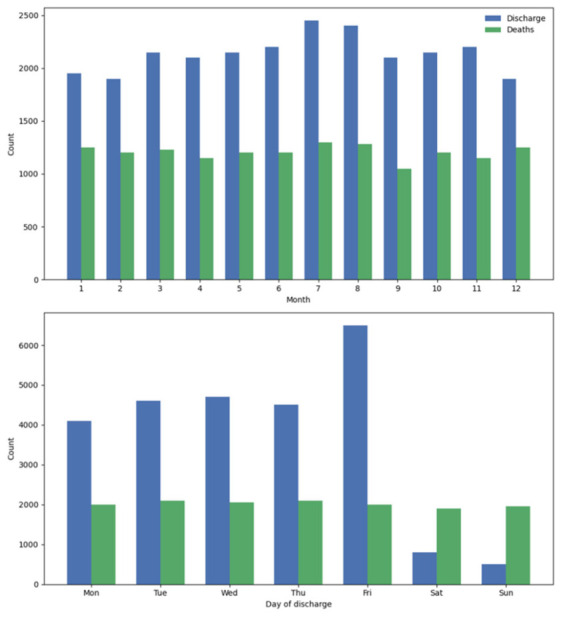
Monthly (upper panel) and weekly (lower panel) distribution of hospital mortality.

Throughout the study period, we observed a significant linear increase in cumulative mortality, accompanied by a statistically significant linear decrease in in-hospital mortality rate: from 42.1% in 2001 to 31.9% in 2021 (*p* < 0.001) (Figure 5). When comparing mortality across the three periods defined by joinpoint analysis, mortality was significantly higher in 2001–2008 (P1) than in 2009–2014 (P2), and higher in P2 than in 2015–2021 (P3). Admission during P1 was an independent risk factor for mortality in multivariable analysis (Figure 4, Table 2).

The months of December, January, July, and August recorded the highest proportion of deaths, with 8.7%, 8.7%, 9.1%, and 9% of deaths, respectively, occurring during these months (*p* < 0.001). These differences were statistically significant despite the absence of a circannual rhythm in cosinor analysis (Figure 5).

No statistically significant differences were observed in mortality according to the day of the week of admission. Although the distribution of deaths was homogeneous across weekdays, there was a non-significant trend toward lower mortality during weekends (Saturday 13.4% and Sunday 13.7%, *p* = 0.051).

#### 2.3.3. Multivariable Logistic Regression

A multivariable logistic regression analysis was performed with in-hospital mortality as the dependent variable. Variables with *p* < 0.1 in univariate analysis were included. Independent variables selected for the final model were age > 75 years, sex, comorbidities (including heart disease, chronic kidney disease, hypertension, liver disease, diabetes, chronic respiratory disease/COPD, neoplasia, alcoholism, smoking status, dyslipidemia), area of residence (urban/rural), hospital level, season of admission, presence of shock, acute kidney injury, need for mechanical ventilation, type of pathogen, presence of bacteremia, and study period (P1: 2001–2008, P2: 2009–2014, and P3: 2015–2021).

Forward stepwise analysis (five steps required) identified the following independent risk factors for in-hospital mortality: age > 75 years (OR = 2.091, 95% CI 1.886–2.319, *p* < 0.001), need for mechanical ventilation (OR = 3.111, 95% CI 2.604–3.716, *p* < 0.001), presence of shock at admission (OR = 1.954, 95% CI 1.764–2.165, *p* < 0.001), acute kidney injury (OR = 1.355, 95% CI 1.235–1.487, *p* < 0.001), history of heart disease (OR = 1.260, 95% CI 1.155–1.375, *p* < 0.001), history of malignancy (OR = 1.586, 95% CI 1.415–1.777, *p* < 0.001), admission to a level 2 hospital (vs. levels 1 or 3) (OR = 1.253, 95% CI 1.146–1.370, *p* < 0.001) and Gram-positive bacterial infection (OR = 1.836, 95% CI 1.672–2.018, *p* < 0.001).

Conversely, independent protective factors were surgical vs. medical sepsis (OR = 0.685, 95% CI 0.501–0.935, *p* < 0.001), admission in autumn versus winter (OR = 0.826, 95% CI 0.730–0.936, *p* = 0.003), admission during P2 (OR = 0.706, 95% CI 0.620–0.805, *p* < 0.001) or P3 (OR = 0.674, 95% CI 0.559–0.812, *p* < 0.001) compared with P1 and history of hypertension (OR = 0.876, 95% CI 0.788–0.973, *p* < 0.013).

## 3. Discussion

Our study provides clinical, chronobiological and epidemiological data from a large healthcare region in northwestern Spain. Several findings merit particular attention. First, we observed a significant increase in the cumulative incidence of sepsis as the principal cause of hospital admission over the 21-year study period. Similar trends have been reported in other studies and have been linked to factors such as population aging, rising antibiotic resistance and the expanding use of immunosuppressive therapies. This finding is consistent with epidemiological data from other countries such as England, where Allen et al. reported a 7.5-fold increase in hospital admissions for sepsis over the past two decades [14].

Second, no reproducible circannual chronobiological pattern was identified. Although seasonal variability was present, the absence of a stable rhythm suggests that temporal fluctuations in sepsis are more likely driven by external environmental factors than by endogenous biological cycles.

The epidemiological analysis in our study suggests that the marked increase in incidence may not be solely attributable to demographic and epidemiological changes but also to evolving diagnostic criteria and coding practices. Two temporal inflection points (joinpoints), in 2008 and 2014, defined three distinct periods with different slopes. The most pronounced change occurred between 2008 and 2014, when the annual percentage change (APC) reached 21.8%. In contrast, from 2014 to 2021 the APC was 4.3%. These periods coincide with milestones in the understanding and dissemination of the sepsis concept. Following the clinical implementation of the Surviving Sepsis Campaign (SSC) in 2005, the 2008 international guidelines and sepsis bundles promoted early recognition and standardized treatment, encouraging the creation of multidisciplinary in-hospital “Sepsis Code” teams [15]. Two key factors may explain the second inflection point in 2014. First, the introduction of the updated Sepsis-3 definitions in 2015, which generated considerable debate and widespread attention in the scientific community, likely influenced clinical recognition and diagnostic criteria. Second, the transition from ICD-9 to ICD-10 coding in 2015 may also have influenced the identification and reporting of sepsis cases. Together, these factors may have led to improved detection, documentation, and recording of sepsis cases over time.

These developments likely contributed to improved case detection, better documentation, and earlier initiation of therapy. They may also explain the continuous decline in hospital mortality and length of stay observed during the study period. In our study, the hospital mortality rate decreased by nearly 10% over 21 years despite a progressive increase in patient age. In fact, multivariable logistic regression analysis showed that admission during periods P2 (2009–2014) and P3 (2015–2021) was independently associated with lower mortality compared with the reference period P1 (2001–2008). This supports the notion that earlier recognition and timely treatment improve outcomes in this time-sensitive condition. Advances in supportive therapies (e.g., ECMO, continuous renal replacement therapy) and new antibiotics targeting multidrug-resistant pathogens have probably also contributed to improved survival. A previous international multicenter study already reported a reduction in mortality of around 11% between 2005 and 2011 [16]. However, few studies have analyzed more recent trends. One such study, published in 2022 by Lorencio-Cárdenas et al., retrospectively analyzed data from public hospitals in Catalonia, another region of Spain, showing both a decrease in mortality (from 27.9% to 19.5%) and a significant increase in the incidence of sepsis (from 144 to 410 cases per 100,000 inhabitants) during the period 2005 to 2019 [17]. These findings are comparable to ours. In another recent multicenter study from Norway, a reduction of about 5% in 30-day mortality was observed among patients admitted with sepsis during the period 2008–2021 [18].

Despite these improvements, overall hospital mortality in our cohort remained high (35%), especially in patients older than 75 years, who accounted for nearly 80% of deaths. Age emerged as one of the main independent predictors of mortality, in line with previous reports [19].

Evidence regarding the chronobiology of sepsis remains limited, particularly regarding quantitative approaches using formal mathematical methods. In our study, unlike in other diseases, we did not find significant circannual rhythms in sepsis admissions. This conclusion was based on complementary approaches: Fast Fourier Transform (FFT) analysis, which decomposes time series into dominant frequencies, and multi-harmonic cosinor models, which fit sinusoidal curves to detect biological rhythms. While widely used in other disciplines, these tools are rarely applied in sepsis research, making this study one of the first to apply these methods in sepsis research.

Regarding mortality, cosinor analysis is specifically designed to detect consistent and reproducible sinusoidal patterns over time (i.e., true biological rhythms with a defined periodicity and stable amplitude). In our study, the non-significant result (*p* = 0.14) indicates the absence of a stable circannual rhythm in sepsis incidence or mortality.

In contrast, the observed excess mortality during winter was identified through categorical seasonal comparisons, which assess differences between predefined time periods (e.g., winter vs. other seasons) without assuming a sinusoidal structure. Therefore, these findings likely reflect irregular or non-periodic temporal variations rather than a consistent biological rhythm. Although no circannual rhythm was detected, descriptive analysis revealed mortality peaks in winter (December–January) and, to a lesser extent, in summer (July–August). After adjustment for age, comorbidities, and clinical variables, warmer seasons (summer and autumn) were independently associated with lower mortality compared with winter. Consistent with previous studies [20], these findings may be explained by seasonal factors such as an increased burden of respiratory infections, higher prevalence of viral co-infections, and greater healthcare system strain during winter months, all of which may contribute to worse outcomes in patients with sepsis. To our knowledge, this is the first study applying FFT and multi-harmonic cosinor methods jointly in sepsis, adding methodological originality and refining the interpretation of seasonality beyond strict biological rhythms.

We also observed higher mortality in level 2 hospitals compared with tertiary (level 3) and small community (level 1) centers. This may reflect differences in case-mix, resource availability and organizational factors. Level 1 hospitals, which lack intensive care units, are likely to manage less severe cases and refer critically ill patients early, introducing a selection bias towards lower-risk populations. Tertiary centers typically have greater access to specialized resources, multidisciplinary teams and higher case volumes, which have been associated with improved outcomes. Level 2 hospitals may represent an intermediate scenario, managing a substantial proportion of moderately to severely ill patients but without the full infrastructure or experience of tertiary centers.

Similar findings have been reported elsewhere, supporting the referral of complex sepsis cases to high-level centers [21].

The apparent protective effect of hypertension and dyslipidemia may reflect selection bias: these patients are more likely to be under regular medical follow-up and therefore to receive earlier diagnosis and treatment. In addition, chronic therapies such as ACE inhibitors or statins, which have endothelial-stabilizing and antioxidant properties, have been proposed as potential adjunctive treatments in sepsis [22].

Patients with medical DRGs had higher mortality than those with surgical DRGs, suggesting a worse prognosis. Medical sepsis is often associated with immunosuppression, nosocomial infections, multidrug-resistant organisms, and prolonged hospital stays, all linked to increased mortality, as confirmed by recent multicenter studies [23,24,25].

Our study has several limitations. Its retrospective nature and reliance on the National Hospital Discharge Database (NHDS), based on ICD-9 and ICD-10 coding, may introduce bias. Coding systems depend on accurate physician documentation and clinical codification, and their sensitivity for sepsis identification is limited [26]. Nevertheless, the NHDS is a widely implemented and standardized system covering nearly the entire hospitalized population in the region and has been extensively used in epidemiological research [27,28,29].

Although the presence of a documented Gram-positive bacteria emerged as an independent predictor of mortality, interpretation should be cautious, since no etiological information was available in nearly 60% of cases. This limitation may affect microbiological characterization; however, it does not affect the chronobiological analyses. Finally, generalizability to other countries may be limited, although similar findings have been reported elsewhere. Further studies of a similar nature would be necessary to confirm our results.

Overall, this study provides a robust analysis of long-term clinical, epidemiological and seasonal trends in sepsis and highlights the need for further international research using quantitative chronobiological methods.

## 4. Materials and Methods

### 4.1. Study Design and Setting

The objective of this retrospective observational study was to perform a descriptive analysis of patients requiring hospital admission with a principal diagnosis of sepsis in public hospitals of Castilla y León (Spain) between 2001 and 2021, and to examine their epidemiological, demographic, and chronobiological characteristics. Cases of nosocomial sepsis acquired during hospitalization were not included.

### 4.2. Data Sources

Data for all patients aged ≥18 years hospitalized with sepsis as the principal diagnosis during this period were obtained from the National Hospital Discharge Database (NHDS; “Conjunto Mínimo Básico de Datos” in Spanish). The NHDS is part of the Health Information System of the Spanish Ministry of Health and is a mandatory anonymized registry that includes discharge summaries from approximately 98% of public and private hospitals in Spain, covering an estimated 99.5% of the hospitalized population. Variables collected included age, sex, need for intensive care, use of mechanical ventilation, presence of acute respiratory distress syndrome, nosocomial infection, sepsis or septic shock, in-hospital mortality, and up to 1420 diagnostic and procedural codes. Patients hospitalized with sepsis were identified using ICD-9-CM codes (2005–2017) and ICD-10-CM codes (2018–2021) (see Supplementary Table S1). These ICD codes are based on the 2001 International Sepsis Definitions Conference [13].

### 4.3. Chronobiological Analysis

We performed a chronobiological analysis aimed at identifying structured, biologically driven temporal patterns (i.e., circannual rhythms), rather than simple temporal fluctuations. To assess changes in trends in the number of hospitalizations per 100,000 inhabitants, we applied joinpoint linear regression analysis. This method tests for statistically significant changes over time by identifying inflection points (join-points). Graphically, joinpoint models applied to the logarithm of the rate generate a sequence of connected segments. The point at which these segments meet is a joinpoint, representing a statistically significant change in trend. Within each segment defined by a joinpoint, an annual percentage change in the trend was calculated using generalized linear models, assuming a Poisson distribution, and the corresponding statistical significance was reported with 95% confidence intervals (95% CI). Differences were considered statistically significant at *p* < 0.05. Trend analysis was performed to determine whether statistically significant changes occurred over time in the rates using joinpoint linear regression (Joinpoint Trend Analysis Software version 6.0.0. provided by US National Cancer Institute, Surveillance Research Program).

Temporal patterns (e.g., annual or seasonal variation) were assessed using spectral analysis with the fast Fourier transform, to detect dominant periodic components, and cosinor analysis method described by Nelson et al., with three harmonically related sinusoids, as detailed by Alberola-López and Martín-Fernández [30], to formally test for the presence of sinusoidal biological rhythms over time.

Analyses were performed using the Matlab^®^ R2014a (8.3.0.52) platform. Annual periodicities were examined with the cosinor (cosine-vector) method, which applies least-squares regression to test whether the distribution of time series data for a given variable fits a cosine curve.

The following terms were applied: (a) rhythm: variability of a pattern oscillating within a given period; (b) period: time required to complete one cycle; (c) cosine curve: sinusoidal curve representing oscillation of a variable; (d) amplitude: half the difference between the maximum and minimum of the cosine curve; (e) acrophase: timing of the maximum value; (f) bathyphase: timing of the minimum value; and (g) circannual: a rhythm with a period of approximately 12 months. The cosinor method tests the null hypothesis of zero amplitude for a chosen period, providing rhythm parameters with confidence intervals if a rhythm is detected. An F test compares the variance explained by the model, and amplitude–acrophase tests allow comparison between rhythms. The null hypothesis was rejected at *p* < 0.05. In this study, the variable analyzed was the standardized monthly number of sepsis cases, modeled with periodicities of 12, 6, and 3 months.

These analytical approaches allow differentiation between random or irregular temporal variations and true underlying rhythmic patterns, which may reflect endogenous biological processes.

### 4.4. Statistical Analysis

Incidence rates and trends over the 21-year period were calculated per 1000 hospitalizations per year and per 100,000 inhabitants. Temporal trends were analyzed using joinpoint regression, which identifies statistically significant changes (joinpoints or inflection points) in the direction of the trend. In the joinpoint regression, age is categorized into 5-year intervals using the annual census population data from Castile and León (obtained from the Spanish National Statistics Institute), and it is additionally adjusted according to the 2013 European standard population by age. Graphically, joinpoint models fitted to the logarithm of the rate yield a sequence of connected segments, with joinpoints indicating significant shifts. For each segment, the annual percentage change was estimated using generalized linear models with a Poisson distribution, and statistical significance was reported.

Categorical variables were expressed as absolute numbers and percentages. Continuous variables were reported as mean ± standard deviation. Normal distribution of continuous variables was assessed with the Kolmogorov–Smirnov test and Q–Q plots. Comparisons between categorical variables were performed with the chi-square or Fisher’s exact test as appropriate, and continuous variables were compared using Student’s t test. Logistic regression models were constructed to estimate predictors of in-hospital mortality. Initially, univariate analyses were performed, followed by multivariable logistic regression with stepwise forward selection to identify independent predictors. Statistical analyses were conducted with IBM SPSS Statistics, Version 30.0 (IBM Corp., Armonk, NY, USA). A *p*-value < 0.05 was considered statistically significant.

## 5. Conclusions

Our findings highlight the increasing burden of sepsis as a public health challenge, particularly in aging populations. Improvements in survival appear linked to enhanced awareness, earlier recognition, and therapeutic advances. However, the persistently high mortality rate, especially among vulnerable subgroups, underscores the need for continued efforts in early diagnosis and management.

Notably, excess mortality was concentrated during specific periods of the year, in the absence of a sustained biological rhythm. These findings suggest that sepsis seasonality may be more strongly influenced by external environmental and healthcare factors than by endogenous biological cycles. Integrating chronobiological models with contextual epidemiology may help anticipate surges in healthcare demand and optimize resource allocation.

Further prospective, multicenter studies across diverse populations are needed to validate these results and deepen our understanding of sepsis chronobiology. Only through a comprehensive understanding of the factors influencing the onset, progression, and lethality of sepsis will it be possible to optimize prevention and management strategies in increasingly complex and aging healthcare systems.

## Figures and Tables

**Table 1:** Demographic, epidemiological, and clinical characteristics of the included patients.

	Total (*n* = 39,622)	Non-Survivors (*n* = 14,152)	Survivors (*n* = 25,470)	*p*
**Male (*n*, %)**	21,919 (55%)	7526 (34.3%)	14,393 (65.7%)	<0.001
**Female (*n*, %)**	17,703 (44.7%)	6626 (37.4%)	11,077 (62.6%)	
**Age (years)**	78.6 (±13.3)	82.1 (±11.1)	77 (±14)	<0.001
**Length of stay (days)**	11.6 (±11.9)	7.9 (±11.2)	13.6 (±11.8)	<0.001
**Level 3 hospital (*n*, %)**	21,716 (54.8%)	7519 (34.6%)	14,197 (65.4%)	<0.001
**Level 2 hospital (*n*, %)**	15,260 (38.5%)	5715 (37.5%)	9545 (62.5%)	
**Level 1 hospital (*n*, %)**	2646 (6.7%)	918 (34.7%)	1728 (65.3%)	
**Urban (*n*, %)**	21,014 (53.2%)	7294 (34.7%)	13,720 (65.3%)	<0.001
**Rural (*n*, %)**	18,515 (46.8%)	6838 (36.9%)	11,678 (63.1%)	
**Emergency admission (*n*, %)**	39,249 (99.1%)	14,029 (35.7%)	25,220 (64.3%)	0.26
**Scheduled admission (*n*, %)**	373 (0.9%)	123 (33%)	250 (67%)	
**Malignancy (*n*, %)**				<0.001
**Yes**	6698 (16.9%)	2644 (39.5%)	4054 (60.5%)	
**No**	32,924 (83.1%)	11,508 (35%)	21,416 (65%)	
**Alcohol (*n*, %)**				<0.001
**Yes**	1297 (3.3%)	390 (30.1%)	907 (69.9%)	
**No**	38,325 (96.7%)	13,762 (35.9%)	24,563 (64.1%)	
**Smoking** **(*n*, %)**				<0.001
**Yes**	3009 (7.6%)	743 (24.7%)	2266 (75.3%)	
**No**	36,613 (92.4%)	13,409 (36.6%)	23,204 (63.4%)	
**Heart disease (*n*, %)**				<0.001
**Yes**	17,684 (44.6%)	7206 (40.7%)	10,478 (59.3%)	
**No**	21,938 (55.4%)	6946 (31.7%)	14,992 (68.3%)	
**Diabetes (*n*, %)**				<0.001
**Yes**	9829 (24.8%)	3385 (34.4%)	6444 (65.6%)	
**No**	29,793 (75.2%)	10,767 (36.1%)	19,026 (63.9%)	
**Dyslipidemia (*n*, %)**				<0.001
**Yes**	6971 (17.6%)	2099 (31.1%)	4872 (69.9%)	
**No**	32,651 (82.4%)	12,053 (36.9%)	20,598 (63.1%)	
**COPD (*n*, %)**				0.06
**Yes**	4772 (12%)	1763 (36.9%)	3009 (63.1%)	
**No**	34,850 (88%)	12,389 (35.5%)	22,461 (64.5%)	
**Liver disease (*n*, %)**				<0.001
**Yes**	1730 (4.4%)	550 (31.8%)	1180 (68.2%)	
**No**	37,892 (95.6%)	13,602 (35.9%)	24,290 (64.1%)	
**HTN (*n*, %)**				<0.001
**Yes**	11,326 (28.6%)	3870 (34.2%)	7456 (65.8%)	
**No**	28,296 (71.4%)	10,282 (36.3%)	18,014 (63.7%)	
**CKD (*n*, %)**				<0.001
**Yes**	7734 (19.5%)	2954 (38.2%)	4780 (61.8%)	
**No**	31,888 (80.5%)	11,198 (35.1%)	20,690 (64.9%)	
**GPB (*n*, %)**	3321 (8.4%)	1005 (30.3%)	2316 (69.7%)	<0.001
**GNB (*n*, %)**	10,521 (26.6%)	2024 (19.2%)	8497 (80.8%)	
**Fungi (*n*, %)**	94 (0.24%)	23 (24.5%)	71 (75.5%)	<0.001
**Viruses (*n*, %)**	633 (1.5%)	169 (28.6%)	422 (71.4%)	<0.001
**Shock (*n*, %)**				<0.001
**Yes**	9187 (23.2%)	4470 (48%)	4711 (51.3%)	
**NO**	30,435 (76.8%)	9776 (68.4%)	20,759 (68.2%)	
**IMV (*n*, %)**				<0.001
**Yes**	1787 (4.5%)	964 (53.9%)	823 (46.1%)	
**No**	37,835 (95.5%)	13,188 (34.9%)	24,647 (65.1%)	
**Bacteremia (*n*, %)**				<0.001
**Yes**	758 (1.9%)	167 (22%)	591 (78%)	
**No**	38,864 (98.1%)	13,985 (36%)	24,879 (64%)	
**AKI (*n*, %)**				<0.001
**Yes**	14,416 (36.4%)	6009 (41.6%)	8407 (58.3%)	
**No**	25,206 (63.6%)	8143 (32.3%)	17,063 (67.7%)	
**Season (*n*, %)**				<0.001
**Winter**	9334 (23.6%)	3632 (38.9%)	5702 (61.1%)	
**Spring**	9858 (24.9%)	3502 (35.5%)	6356 (64.5%)	
**Summer**	10,744 (27.1%)	3735 (34.8%)	7009 (65.2%)	
**Autumn**	9686 (24.4%)	3283 (33.9%)	6403 (66.1%)	
**Period 1**	4430 (11.2%)	1925 (43.5%)	3505 (56.5%)	<0.001
**Period 2**	10,754 (27.1%)	3978 (37%)	6776 (63%)	
**Period 3**	24,438 (61.7%)	8249 (33.8%)	16,186 (66.2%)	

The second column shows the total number of patients with each characteristic listed in the first column (absolute values and percentages relative to the entire cohort). The third and fourth columns show the proportion of patients within each category classified as deceased or survivor, respectively. Continuous variables are reported as mean ± standard deviation. Abbreviations: COPD: Chronic obstructive pulmonary disease; HTN: Hypertension; CKD: Chronic kidney disease; GPB: Gram-positive bacteria; GNB: Gram-negative bacteria, AKI: Acute kidney injury; IMV: Invasive mechanical ventilation.

**Table 2:** Multivariate logistic regression analysis for hospital mortality.

Variables	*p*	OR	95%CI	
Age > 75 years	<0.001	2.091	1.886	2.319
Female sex	0.071	1.083	0.993	1.181
Heart disease	<0.001	1.26	1.155	1.375
HTN	<0.013	0.876	0.788	0.973
Malignancy	<0.001	1.586	1.415	1.777
CKD	0.005	1.177	1.052	1.318
Dyslipidemia	<0.001	0.649	0.567	0.742
Shock	<0.001	1.954	1.764	2.165
IMV	<0.001	3.111	2.604	3.716
AKI	<0.001	1.355	1.235	1.487
Hospital level 2	<0.001	1.253	1.146	1.37
Surgical vs. Medical DRG	<0.001	0.685	0.501	0.935
Rural area	0.06	1.087	0.996	1.185
GPB	<0.001	1.836	1.672	2.018
Spring vs. winter	0.218	0.933	0.827	1.053
Summer vs. winter	0.047	0.822	0.726	0.931
Autumn vs. winter	0.003	0.826	0.73	0.936
P2 vs. P1	<0.001	0.706	0.62	0.805
P3 vs. P1	<0.001	0.674	0.559	0.812

HTN: hypertension; CKD: chronic kidney disease; IMV: invasive mechanical ventilation; AKI: acute kidney injury; GPB: Gram-positive bacteria; P1: period 2001–2008; P2: period 2009–2013; P3: period 2014–2021.

## Data Availability

The data that support the findings of this study are available from the corresponding author upon reasonable request.

## Supplementary Materials

The following supporting information can be downloaded at https://www.mdpi.com/article/10.3390/antibiotics15060579/s1: Figure S1: Distribution of patients by hospital type (left) and DRG type (right); Table S1: ICD 9 and 10 codes used to define the condition of sepsis or septic shock.
