# Supplementary material for: Temporal Trends in the Chronobiology and Epidemiology of Sepsis Admissions over 21 Years: A Nationwide Study in Northwestern Spain
Source: Antibiotics (Basel). 2026 Jun 7;15(6):579. doi: 10.3390/antibiotics15060579 (PMC13295478; doi:10.3390/antibiotics15060579)
Supplement: Supplementary file 1 [file antibiotics-15-00579-s001.zip › antibiotics-4201053-supplementary.pdf]

Supplementary Table S1. ICD 9 and 10 codes used to define the condition of sepsis or septic shock.

**Entity**	**ICD-9 code**	**ICD-10 code**
**Sepsis**	038.xx, 995.91	A40, A41, A39.2-A.39.4, A32.7, B00.7
**Severe Sepsis**	995.92	R65.20
**Septic Shock**	785.52	R65.21
**Sepsis Gram +**	038.0, 038.1	A40, A41.0, A41.1, A41.2, A41.81,
**Sepsis Gram -**	038.4, 038.40–038.49	A41.3, A41.5, A39-2-A39.4, A54.86
**Sepsis Anaerobics**	038.3	A41.4
**Sepsis virus**	079.99 + 995.91 or 995.92	B007+ A40, A41 , R65.20 or R65.21

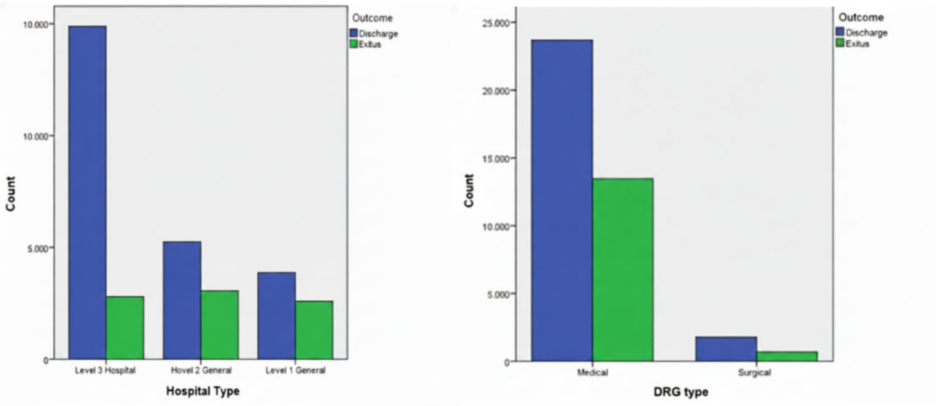

Supplementary Figure S1. Distribution of patients by hospital type (left) and DRG type (right).
